# Association between long-term exposure to ambient air pollution and prevalence of diabetes mellitus among Malaysian adults

**DOI:** 10.1186/s12940-020-00579-w

**Published:** 2020-04-03

**Authors:** Shew Fung Wong, Poh Sin Yap, Joon Wah Mak, Wan Ling Elaine Chan, Geok Lin Khor, Stephen Ambu, Wan Loy Chu, Maria Safura Mohamad, Norazizah Ibrahim Wong, Nur Liana Ab. Majid, Hamizatul Akmal Abd. Hamid, Wan Shakira Rodzlan Hasani, Muhammad Fadhli bin Mohd Yussoff, Hj. Tahir bin Aris, Ezahtulsyahreen Bt. Ab. Rahman, Zaleha Bt. M. Rashid

**Affiliations:** 1 Institute for Research, Development and Innovation (IRDI), International Medical University, 57000 Kuala Lumpur, Malaysia; 2 School of Medicine, International Medical University, 57000 Kuala Lumpur, Malaysia; 3 School of Postgraduate Studies, International Medical University, 57000 Kuala Lumpur, Malaysia; 4 Institute for Public Health, Ministry of Health, 40170 Shah Alam, Selangor Malaysia; 5 Department of Environment, Ministry of Energy, Technology, Science, Environment and Climate Change, 62662 Putrajaya, Malaysia

**Keywords:** Nitrogen dioxide (NO_2_), Sulphur dioxide (SO_2_), Ozone (O_3_), Particular matters (PM_10_), Air pollution, Diabetes

## Abstract

**Background:**

Malaysia has the highest rate of diabetes mellitus (DM) in the Southeast Asian region, and has ongoing air pollution and periodic haze exposure.

**Methods:**

Diabetes data were derived from the Malaysian National Health and Morbidity Surveys conducted in 2006, 2011 and 2015. The air pollution data (NO_x_, NO_2_, SO_2_, O_3_ and PM_10_) were obtained from the Department of Environment Malaysia. Using multiple logistic and linear regression models, the association between long-term exposure to these pollutants and prevalence of diabetes among Malaysian adults was evaluated.

**Results:**

The PM_10_ concentration decreased from 2006 to 2014, followed by an increase in 2015. Levels of NO_x_ decreased while O_3_ increased annually. The air pollutant levels based on individual modelled air pollution exposure as measured by the nearest monitoring station were higher than the annual averages of the five pollutants present in the ambient air. The prevalence of overall diabetes increased from 11.4% in 2006 to 21.2% in 2015. The prevalence of known diabetes, underdiagnosed diabetes, overweight and obesity also increased over these years. There were significant positive effect estimates of known diabetes at 1.125 (95% CI, 1.042, 1.213) for PM_10_, 1.553 (95% CI, 1.328, 1.816) for O_3_, 1.271 (95% CI, 1.088, 1.486) for SO_2_, 1.124 (95% CI, 1.048, 1.207) for NO_2_, and 1.087 (95% CI, 1.024, 1.153) for NO_x_ for NHMS 2006. The adjusted annual average levels of PM_10_ [1.187 (95% CI, 1.088, 1.294)], O_3_ [1.701 (95% CI, 1.387, 2.086)], NO_2_ [1.120 (95% CI, 1.026, 1.222)] and NO_x_ [1.110 (95% CI, 1.028, 1.199)] increased significantly from NHMS 2006 to NHMS 2011 for overall diabetes. This was followed by a significant decreasing trend from NHMS 2011 to 2015 [0.911 for NO_2_, and 0.910 for NO_x_].

**Conclusion:**

The findings of this study suggest that long-term exposure to O_3_ is an important associated factor of underdiagnosed DM risk in Malaysia. PM_10_, NO_2_ and NO_x_ may have mixed effect estimates towards the risk of DM, and their roles should be further investigated with other interaction models. Policy and intervention measures should be taken to reduce air pollution in Malaysia.

## Introduction

According to the International Diabetes Federation (IDF) Western Pacific statistics, 3.5 million cases of diabetes mellitus type 2 (T2DM) were reported in Malaysia in 2017, and the prevalence rate in adults (20–79 years) was 16.9% [1]. The prevalence of T2DM among Malaysian adults has increased from 6.3% in 1986 to 17.5% in 2015 [2, 3]. During this period of time, the prevalence of overweight and obesity among Malaysians has escalated [4], in tandem with the global situation.

T2DM has been attributed to several risk factors. Among these, genetics, lifestyles, or in combination have been extensively studied [5, 6]. Environmental factors that have been linked to T2DM include air pollution, unhealthy diet and lack of physical activity [7, 8].

Environmental stimuli such as pollutants can activate intracellular signalling pathways which alter the gene expression through epigenetic modifications such as DNA methylation and histone post-translational modifications. In addition, exposure to higher ambient levels of pollutants such as NO_2_ and PM_2.5_ was found to be associated with lower and declined insulin sensitivity, increased β-cell dysfunction and increased adipose deposition or obesity [9, 10].

The association between air pollution and the development of diabetes has been reported worldwide. In Switzerland, Eze et al. [11] found that both long-term exposure to PM_10_ and NO_2_ even at the concentrations below the air quality guidelines was associated with increased prevalence of diabetes mellitus in a cohort study [11]. Another study in Germany showed that individuals with higher genetic risk for diabetes, such as those with altered insulin sensitivity, were more susceptible to the exposure of PM_10_ where the odds of diabetes increased by 8% per risk allele and 35% per 10 μg/m^3^ exposure to PM_10_ [12]. In addition, hazards of diabetes were found to increase by 15–42% per interquartile range (IQR) of traffic-related particular matters and NO_2_ exposure in elderly women in a study conducted in West Germany [13].

Ambient air pollution increased mortality resulting from respiratory and cardiovascular diseases as well as diabetes mellitus [14]. Urban traffic is one of the major sources of ambient air pollution, followed by industrial and biomass burning. All these sources release fine particles and gaseous pollutants into the atmosphere which will eventually induce systemic oxidative stress and inflammatory responses [14]. Malaysia is one of the developing countries with high levels of outdoor and indoor air pollution from motor vehicles, industries and biomass. Since 1991, haze largely due to fire-related air pollution occurs regularly subsequent to large-scale agricultural and forest burning in the region [15]. All these sources release particulate matters such as toxic metals, carbon monoxide, oxides of sulphur and nitrogen, which are harmful to human health and the ecosystem. Several studies have reported short-term adverse effects of air pollution on public health during these haze periods [16–19]. Exposure to haze and air pollutants may cause irritation to eyes, nose and throat, eczema, allergy, asthma, bronchitis and other systemic health problems including cardiovascular diseases.

Emissions from motor-vehicles and industries in Asian countries are under-regulated, compared with Western countries [20]. The situation is further worsened by trans-boundary haze associated with biomass burning. To date, limited studies have been conducted in Malaysia that assessed adverse health impacts from exposure to air pollutants. We aimed to determine the association between exposure to air pollution and prevalence of diabetes mellitus in the Malaysian population.

## Methods

### Study population

Data on status including diabetes, body mass index and physical activity reported in this study were derived from three national cross-sectional health surveys, namely the National Health and Morbidity Surveys undertaken in 2006, 2011 and 2015 [21–23]. Each survey included representative samples of men and women throughout Malaysia. The representative samples were derived from a 2-stage stratified random sampling of the population based on the proportionate of the population size as determined using the Enumeration Blocks (EBs, made up of states of Malaysia including Federal Territories), followed by Living Quarters (LQs, made up of urban/rural areas). The urban area is gazette area with a combined population of 10,000 or more at the time of census while the rural area is with less than 10,000 individuals. Each EB had an average of 100 LQ while each LQ was estimated to house 4.4 individuals. All individuals of the selected LQ were included for the survey after obtaining their consents. The sampling frame was recommended by Department of Statistics, Malaysia. Details of the NHMS study’s methodology has been published elsewhere [21–23] (http://iku.moh.gov.my/index.php). Informed consent was obtained from each participant. Thumb print was taken from illiterate subjects in the presence of a witness. The NHMS surveys were approved by the Malaysian Medical and Research Ethnic Committee, Ministry of Health Malaysia (NMRR-16-424-29,188 (IIR)). The present study was approved by the International Medical University Joint-Committee on Research and Ethics (reference no: 4.11/JCM-110/2016).

While the NHMS data included Malaysians of all ages, participants aged 17 and below were excluded from this analysis in order to exclude Type 1 diabetes in the present study. In Malaysia, most Type 1 diabetes (> 70%) patients were diagnosed at ages below 15 years [24]. Social-demographic information was gathered from consented respondents by a trained nurse using a structured questionnaire. Fasting blood glucose of each respondent was determined. The prevalence of known diabetes was determined by asking the participants whether they had been medically diagnosed with diabetes. Underdiagnosed diabetes cases reported in the three surveys refer to those participants who are not aware that they are having diabetes and were identified based on the definition of the diagnostic criteria of the World Health Organisation for fasting capillary blood glucose equal or more than 6.1 mmol/L, or non-fasting glucose of more than 11.1 mmol/L [25]. Overall diabetes prevalence was calculated as the sum of known diabetes and the underdiagnosed diabetes.

Participants were determined as active when the physical activity achieved a minimum of at least 600 Metabolic Equivalent of Task (MET)-minutes per week according to the Global Physical Activity Questionnaire (GPAQ) for year 2006, and the International Physical Activity Questionnaire (IPAQ) for years 2011 and 2015. As for body weight status, body mass index (BMI) as classified according to the World Health Organisation [26] was used: underweight: < 18.5 kg/m^3^; normal: BMI 18.5–24.9 kg/m^3^; overweight: 25.0–29.9 kg/m^3^; and obese: 30.0 kg/m^3^ and above.

### Air pollution data

The daily air pollution data on nitrogen oxide (NO_x_), nitrogen dioxide (NO_2_), sulphur dioxide (SO_2_), ozone (O_3_), and particulate matters less than 10 μg/m^3^ (PM_10_) were obtained from the Department of Environment Malaysia (DOE). These five ambient air pollution data recorded by the Continuous Air Quality Monitoring (CAQM) stations were collected from 2006 to 2015 in all the 13 states and three Federal Territories of Malaysia. There are 52 monitoring stations which are strategically located at different industrial (25%), urban (29%), suburban (38%) and background areas (8%) respectively in Malaysia [27]. The monitoring stations at industrial sites are located in almost every state of Malaysia except for Perlis, Kedah, Sabah, and three Federal Territories of Malaysia i.e. Putrajaya, Kuala Lumpur and Labuan.

Data of the five ambient air pollution parameters were recorded hourly per day. For states with more than one monitoring station, the annual averages of the five ambient air pollution parameters were calculated using the averages across all monitoring stations within the same state. Air monitoring stations with more than 25% missing records per year were excluded from the annual calculation of the five ambient air pollution parameters for the same state or Federal Territory. The overall pollution exposure for each participant was calculated as the average level of exposure over the year and was assigned to the participants of the three NHMS surveys based on the air monitoring station that is most adjacent to the residential coordinate. In those states with multiple monitoring stations, an average reading per parameter was presented as a whole in this study. As for the individual exposure, each individual was matched to the nearest monitoring station data points/parameters during the analysis based on the coordinates (latitude and longitude) of the residential address and monitoring station.

### Statistical analysis

Data was analysed using Statistical Analysis Software (version 9.3 SAS Institute Inc., Cary, NC). Since the NHMS survey has a complex design, PROC SURVEYFREQ and PROC SURVERYLOGISTIC procedures along with sample weights were performed to assess the prevalence of overall diabetes, known diabetes, and underdiagnosed diabetes.

The model used to assign the baseline individual exposure was determined using a different cohort of consented subjects in 1996 based on their postal codes, regardless of the data availability in other years. That cohort had similar age distribution and proportion of immigrants as the current cohort of this study (results not shown). Here, the associations of O_3_, PM_10_ and NO_2_ with non-accidental mortality were nearly identical (i.e. 1.034, 95% CI: 1.028, 1.039); similar but with attenuated associations (i.e., 1.025; 95% CI: 1.020, 1.031); and notably weaker evidence of an association (i.e., 1.017; 95% CI: 1.009, 1.025) respectively to those reported in the main models of this study.

Logistic regression models were used to adjust for the following predictors: age, ethnicity, urban and rural, gender, and the 2006, 2011 and 2015 annual averages of air pollution for NO_x_, NO_2_, SO_2_, O_3_, and PM_10_ per IQR increment of 16.83 μg/m^3^, 9.57 μg/m^3^, 4.16 μg/m^3^, 7.83 μg/m^3^, and 10.34 μg/m^3^ respectively. Significant interactions in terms of survey year and each air pollution measurement from the logistic models were assessed to determine if changes in the prevalence of diabetes occurred throughout the nine-year period. The adjusted odds ratios (OR) and 95% confidence intervals (CI) of overall diabetes, known diabetes and underdiagnosed diabetes over time according to annual average air pollution levels were calculated.

## Results

**Fig. 1:**
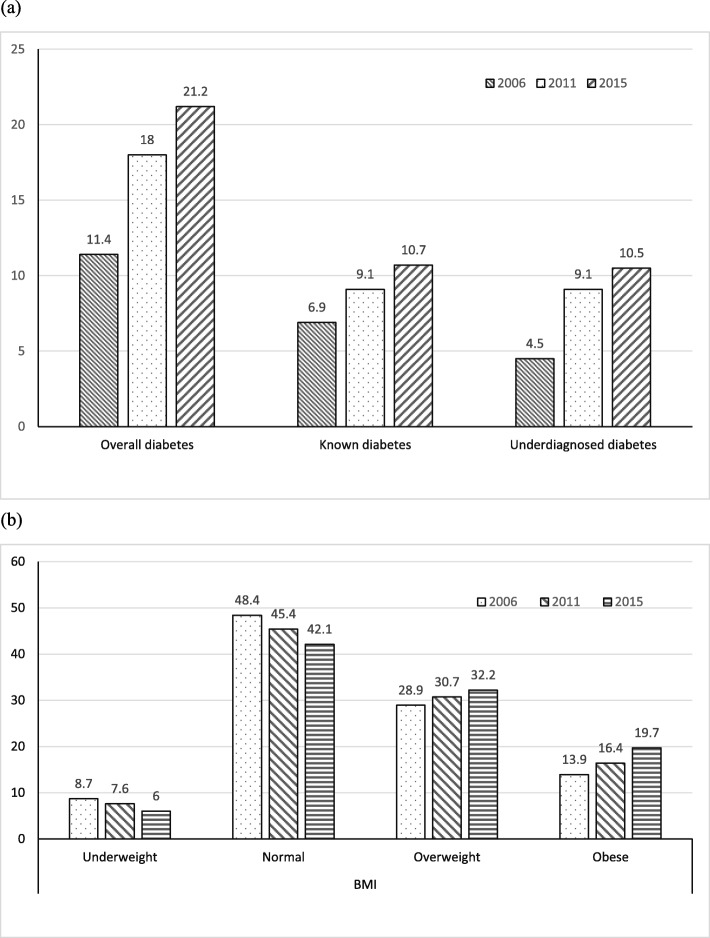
Prevalence of (**a**) overall diabetes, known diabetes and underdiagnosed diabetes, and (**b**) underweight, normal, overweight and obese by NHMS survey years

The characteristics of the respondents included in the three NHMS surveys are presented in Table 1. Overall, the distributions of age, sex and ethnicity were relatively similar across these surveys. The prevalence of overall diabetes for all age groups increased from 11.4% in 2006 to 21.2% in 2015 (Fig. 1a). Similarly, the prevalence of known diabetes and underdiagnosed diabetes increased across the surveys (Fig. 1a). The prevalence of overweight and obesity also showed an increase during the surveys (Fig. 1b).

**Table 1:** Characteristics of the respondents included in this study

Characteristics	NHMS 2006(***N*** = 56,710)	NHMS 2011(***N*** = 28,498)	NHMS 2015(***N*** = 29,460)
**Strata**			
Urban	32,734 (57.7%)	16,372 (57.4%)	16,880 (57.3%)
Rural	23,976 (42.3%)	12,126 (42.6%)	12,580 (42.7%)
^a^**Age Group**			
0–7	9988 (17.6%)	4426 (15.5%)	4303 (14.6%)
8–17	12,143 (21.4%)	5841 (20.5%)	5198 (17.6%)
18–24	5587 (9.9%)	2893 (10.2%)	2733 (9.3%)
25–34	7333 (12.9%)	3986 (14.0%)	4272 (14.5%)
35–44	7530 (13.3%)	3643 (12.8%)	3712 (12.6%)
45–54	6618 (11.7%)	3482 (12.2%)	3766 (12.8%)
55–64	4197 (7.4%)	2460 (8.6%)	3027 (10.3%)
65+	3274 (5.8%)	1767 (6.2%)	2449 (8.3%)
**Gender**			
Male	26,709 (47.1%)	13,757 (48.3%)	14,225 (48.3%)
Female	30,001 (52.9%)	14,741 (51.7%)	15,235 (51.7%)
**Ethnicity**			
Malays	32,493 (57.3%)	16,975 (59.6%)	18,845 (64.0%)
Chinese	10,251 (18.1%)	4944 (17.3%)	4284 (14.5%)
Indian	4415 (7.8%)	2122 (7.4%)	1993 (6.8%)
Indigenous population of Malaysia	7093 (12.5%)	2933 (10.3%)	2737 (9.3%)
Others	2458 (4.3%)	1524 (5.3%)	1601 (5.4%)
^b^**Physical Activities**			
Active	19,246 (56.7%)	12,196 (63.7%)	13,872 (67.1%)
Inactive	14,690 (43.3%)	6949 (36.3%)	6804 (32.9%)

*NHMS*: National Health Morbidity SurveyEstimates are weighted to be representative of Malaysian population

^a^indicates missing data of 40 for year 2006

^b^indicates missing data of 22,774, 10,718 and 8784 for year 2006, 2011 and 2015 respectively

**Fig. 2:**
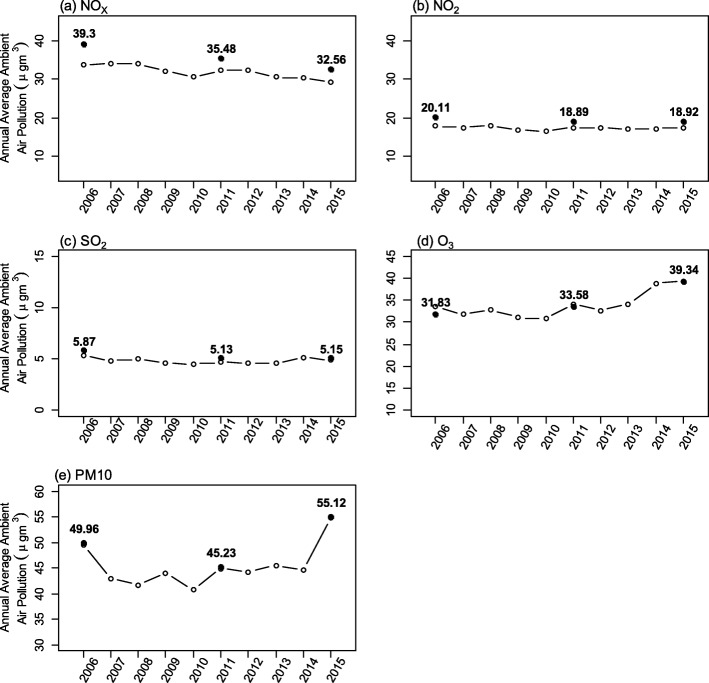
Annual average levels of **a** NO_x_, **b** NO_2_, **c** SO_2_, **d** O_3_ and **e** PM_10_ in ambient air of Malaysia from 2006 to 2015. Note: solid black circles denote individual exposure levels to these pollutants (NO_x_, NO_2_, SO_2_, O_3_ and PM_10_) during the survey years while transparent circles denote annual averages of the five pollutants present in the ambient air

The annual average levels of NO_x_, NO_2_, SO_2_, O_3_ and PM_10_ in the different states and Federal Territories from the year 1996 to 2015 are presented in Supplementary Fig. 1. The annual average levels of NO_x_, NO_2_, SO_2_, O_3_ and PM_10_ from 2006 to 2015 and during the NHMS surveys are presented in Fig. 2. PM_10_ levels fluctuated over the years whereby its level decreased by 9.5% from 2006 to 2011, but increased by 21.8% in 2015. The changes in the levels of ambient air pollutants (NO_2_ and SO_2_) showed similar trends over the last 9 years, whereby a slight decrease was shown from 2006 to 2014, followed by an increase in 2015. The NO_x_ levels decreased between 2006 and 2015 from 39.3 to 32.56 μg/m^3^, while the O_3_ levels increased annually from 2006 to 2015. In general, the individual exposure levels to these pollutants (NO_x_, NO_2_, SO_2_, O_3_ and PM_10_) were higher than the annual averages of the five pollutants present in the ambient air.

**Fig. 3:**
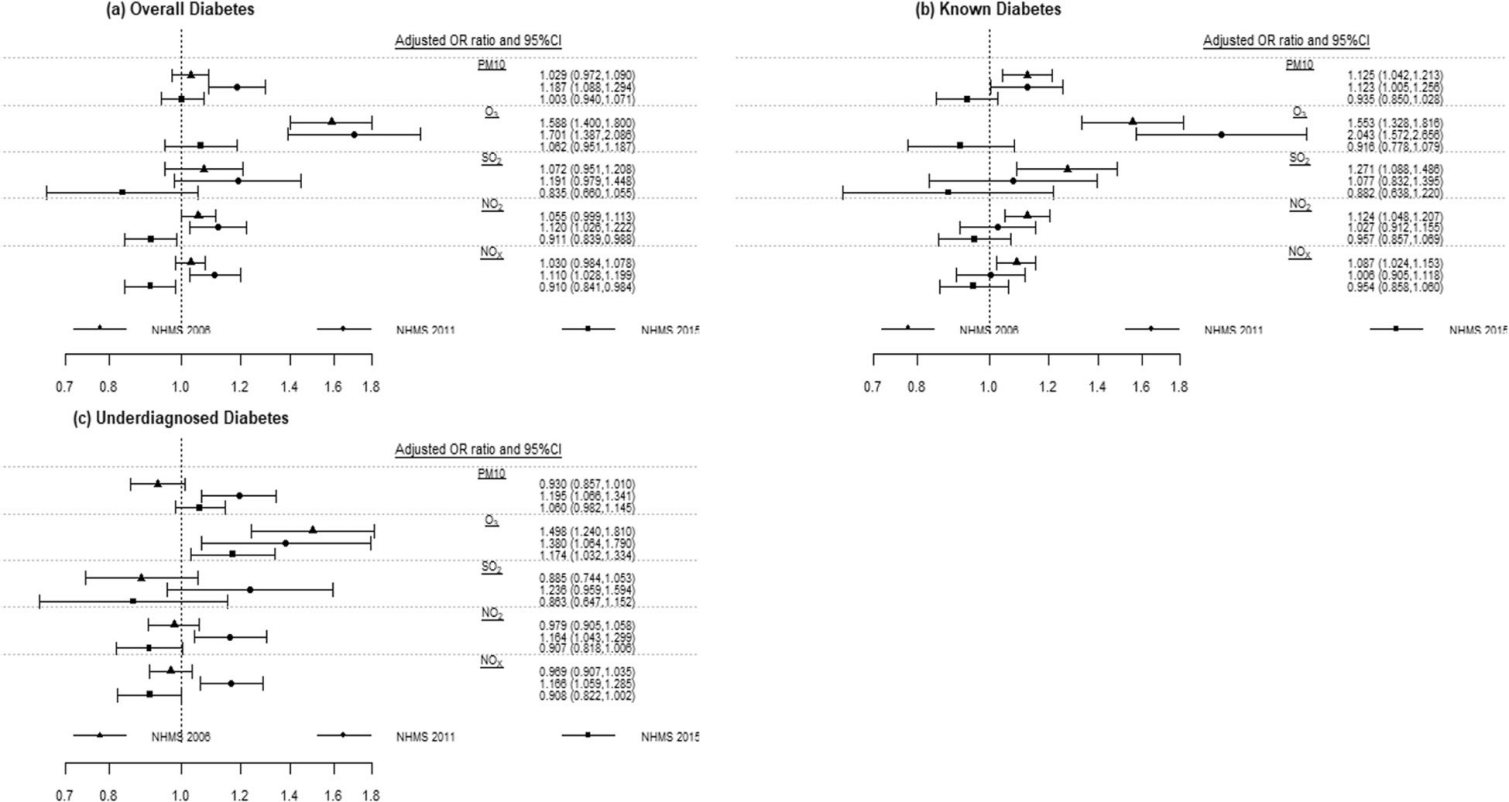
Odds ratio and 95% CIs between diabetes and annual average levels for air pollution exposures by NHMS survey years. Note: The ambient air pollution was modelled with the interquartile range increment (IQR) for the annual average levels of air pollutant exposures: IQR of NO_x_ = 16.83 μg/m^3^; NO_2_ = 9.57 μg/m^3^; SO_2_ = 4.16 μg/m^3^; O_3_ = 7.83 μg/m^3^; PM_10_ = 10.34 μg/m^3^. The models were adjusted for age, gender, ethnicity, urban/rural areas, body mass index (BMI) and physical activity

The adjusted ORs for the annual average levels of all five air pollution exposures over the periods from 2006 to 2015 are presented in Fig. 3. For the overall diabetes, interesting patterns were observed. The adjusted ORs per IQR increment levels (μg/m^3^) of the annual average levels of all five air pollutants increased from NHMS 2006 to 2011, followed by a decreasing trend from NHMS 2011 to 2015 (Fig. 3a). A significant positive interaction effect was observed between exposure to O_3_ and NHMS 2006 (OR = 1.187, 95% CI, 1.088, 1.294). Positive interaction terms between the NHMS 2011 and the four annual average levels of air pollution exposures were statistically significant (*p* < 0.05) (except for SO_2_). However, none of the interaction terms for the NHMS 2015 and the annual average levels of all three air pollutants (PM_10_, O_3_ and SO_2_) showed statistical significance at 0.05 level, except for NO_2_ and NO_x_.

Different results were observed for the known diabetes (Fig. 3b), whereby the adjusted ORs decreased over the NHMS surveys in 2006, 2011 to 2015 except for O_3_ in NHMS 2011. Statistically significant interaction terms were observed for all the five annual average air pollution exposure levels in 2006, while those for O_3_ and PM_10_ were found only in NHMS 2011. No statistical significance was observed between the interaction terms of the annual average levels of all the five air pollutants in NHMS 2015.

The adjusted ORs per IQR increment levels of each air pollutant for underdiagnosed diabetes showed similar patterns as overall diabetes, increasing from NHMS 2006 to NHMS 2011 (except for O_3_), followed by a decrease from NHMS 2011 to NHMS 2015 (Fig. 3c). However, statistically significant (*p* < 0.05) interaction terms between NMHS 2011 and the annual average levels for all the five air pollutant levels for overall diabetes were different from those for underdiagnosed diabetes. The positive interaction terms with the three NMHS and annual average levels for O_3_ exposure were statistically different (p < 0.05). Significant positive associations of underdiagnosed diabetes with PM_10_, O_3_, NO_2_ and NO_x_ were also found in the NHMS 2011.

## Discussion

This is the first study, to the best of our knowledge that investigated the long-term association between ambient air pollution exposure and the prevalence of diabetes in Malaysia over a duration of 9 years (2006–2015).

The WHO air quality guidelines recommended that particulate matters should be below 10 and 20 μg/m^3^ (or 25 and 50 μg/m^3^ 24-h mean) for PM_2.5_ and PM_10_ respectively. Particulate matters include both organic and inorganic suspended solid and liquid particles. Sulphates, nitrates, ammonia, sodium chloride, black carbon and mineral dust are the components found to be associated with short- and long-term health impacts. The present study showed that exposure to PM_10_ in Malaysia was significantly associated with higher prevalence of known diabetes for years 2006 and 2011. However, this association was not seen in 2015 despite the highest PM_10_ recorded during that year. This could be due to the presence of bias or delay effect during the exposure assessment, outcome assessment and confounder assessment as specified by O’Donovan et al. [28]. In order to reduce exposure bias, standard prevailing estimates of outdoor particulate matters and nitrogen oxide concentration were confined to an area within 1 × 1 km of the volunteer’s house postcode in that particular study. Unfortunately, the exposure of individual volunteers in the present study was based on the concentrations of these pollutants recorded by the most adjacent monitoring station of different coverage sizes. Exposure to air pollution was based on residential location and may not reflect actual exposure. One option to tackle this would be to only include individuals that live close to a monitoring station (e.g. within 10, 50 or 100 km) in a sensitivity analysis. A sensitivity analysis to exclude those who are living outside 50 km radius from the air monitoring station was performed (supplementary Fig. 2) and only slight differences were noticed (OR of SO_2_ for overall diabetes of NHMS 2015, OR of PM_10_ for known diabetes of NHMS 2011, and ORs of O_3_, NO_2_ and NO_x_ for underdiagnosed diabetes of NHMS 2015). Hence, exposure measurement error may contribute little impact to the observed association in this study. Other unknown factors may have contributed to the observed association. Another limitation of the study is that the onset of diabetes for each subject was not available for more accurate analysis. In addition, the duration of diabetes, what diabetes medication prescription, whether those with diabetes are compliance to medication and lifestyle changes and etc. are not captured in the present study.

The fasting capillary blood glucose of each volunteer in the present study was measured with a portable whole blood test system and categorised as having diabetes when the concentration is ≥6.1 mmol/L or non-fasting glucose of ≥11.0 mmol/L [25]. This approach is different from that used by O’Donovan et al. [28], which included volunteers with fasting glucose ≥7.0 mmol/L or two-hour glucose ≥11.0 mmol/L using oral glucose tolerance test. There were other limitations in the present study due to confounders such as smoking, area social deprivation, noise pollution, indoor air pollution, and neighbourhood green space, which have not been adjusted accordingly in the analysis, compared to the previous study by O’Donovan et al. [28].

The association of particulate matters with the development of diabetes is mixed ranging from strong, weak to no association [13, 29–34]. Many groups investigated the exposure to PM_2.5_ or combination of PM_2.5_ and PM_10_ instead of PM_10_ alone, as analysed in the present study. The travel distance of coarse PM_10_ ranges from 1 to 10 km while fine PM_2.5_ ranges from 100 to 1000 km from the source of origin. The fine particles can be suspended for a longer period in the air (days to weeks) than the coarse particles in hours [35]. The size of the particles will affect their penetration capacity into the lungs. While the coarse particles can be easily eliminated by muco-ciliary expulsion, sneezing and coughing, the fine particles can easily enter bronchioles and alveoli, and can even penetrate the lungs and enter the blood stream directly. Hence, measurement of PM_10_ concentration and exposure which may include ultrafine, fine and course particles in the present study will limit the determination of causal effect of these particles and disease occurrence.

In the present study, a 10.34 μg/m^3^ increase of PM_10_ in NHMS 2011 led to 19% significant increase in overall diabetes in Malaysia while not for NHMS 2006 and 2015 (3 and 0.3% respectively). Several studies have reported the association between PM_2.5_, instead of PM_10_ and the prevalence of diabetes. For instance, an increase of 19% in NHMS 2011 was relatively higher than that reported by Pearson et al. [36] at 1% increase in diabetes prevalence with a 10 μg/m^3^ increase of PM_2.5_ in the United States. Similarly, in Canada, 10 μg/m^3^ increases in PM_2.5_ were associated with an increase in diabetes mortality (hazard ratio: 1.49; 95% CI: 1.37–1.62) [30]. Another study in Ontario Canada reported hazard ratio of 1.08 for diabetes for every 10 μg/m^3^ increase of PM_2.5_ after gender, age, survey year and region adjustment [31]. Increase in PM_2.5_ has also been shown to be associated with increased diabetes prevalence in China [37]. There was at least one study conducted in the United States that included PM of various sizes in relation to diabetes incidence [32]. The study showed that there was no strong evidence of association between exposure to PM_2.5_, PM_10_ and PM_2.5–10_ in the previous 12 months and the incident of type 2 diabetes among women of two prospective cohorts of the Nurses’ Health Study (NHS) and the Health Professionals Follow-Up Study (HPFS) [32].

The mechanistic explanation for the increase of diabetes prevalence due to PM, as found in the present study, could be due to their effects on the inflammatory reactions. For instance, PM were reported to have inverse association with DNA methylation of the inflammatory genes (e.g. CD14 and Toll-like receptor 4) in peripheral blood of healthy overweight/obese subjects [38]. CD14 is an inflammatory cytokine produced by monocytes/macrophages and serves an innate immune receptor against lipopolysaccharide of microorganism. CD14 together with its downstream signalling Toll-like receptor (TLR) 4 will lead to the activation of the nuclear factor κB inflammatory cascade which eventually stimulates the production of pro-inflammatory cytokines like tumour necrosis factor (TNF). Hence, subsequent to long term exposure to particulate matters might trigger excessive inflammatory responses. Similarly, PM_2.5_ was reported to be associated with higher fasting blood glucose among non-diabetic individuals. PM_2.5_ reduced the blood intercellular adhesion molecule-1 (ICAM-1) methylation which eventually up-regulates the expression of ICAM-1 (an inflammatory cytokine) on leukocytes [39]. In addition, a 10 μg/m^3^ increase of both the fine and coarse particulate matters was significantly associated with 18% increase fractional exhaled nitric oxide (FeNO, a non-invasive biomarker of airway inflammation; 95% CI: 0.18, 2.20) among diabetes patients [40] and decreased endothelial function among individuals who are free of clinical cardiovascular disease [41].

Ambient ozone in the present study was significantly associated with 59–70% increase of overall diabetes in 2006 and 2011. Individual exposure to O_3_ level increased throughout the survey years. On average, the odds of diabetes increased approximately 50% per IQR increment of O_3_. It is noteworthy that the concentration of O_3_ in the ambient air of Malaysia increased throughout the survey years. Ozone is a hazardous photochemical pollutant formed after reaction of the volatile organic compounds with NO_x_ (NO, NO_2_) emitted by industries and automobile exhausts in the present of UV rays. The trapped ozone in the urban areas was associated with 18% increase of diabetes incidence (95% CI: 1.04, 1.34) of African American women [42]. Ozone induced whole-body insulin resistance and oxidative stress, endoplasmic reticulum stress, c-Jun N-terminal kinase (JNK) activation and impaired insulin signalling in skeletal muscle of the Wistar rat model [43]. Renzi et al. [44] reported an association between O_3_ and diabetes incidence [hazard ratio of 1.015 (1.002–1.027) per 10 μg/m^3^ increases] among 1,425,580 subjects aged 35 and above in Rome Italy, and this association was stronger in women than men and those aged < 50 years. Bass et al. [45] suggested that O_3_ could induce adverse systemic metabolic impairments (increased α2-macroglobulin, adiponectin, osteopontin, leptin, endoplasmic reticulum stress and decreased phosphorylation of insulin receptor substrate-1 in liver and adipose tissues) of brown Norway rats via sympathetic nervous system stimulation. This study provided a new epidemiological evidence to support the above mentioned animal studies on the possible adverse effect of long term O_3_ in causing diabetes.

Results showed that there were significant positive associations between nitrogen oxides (NO_2_) and NO_x_ exposures and the prevalence of overall diabetes for 2011 while significant negative interaction term between NO_2_ / NO_x_ and overall diabetes for 2015. No significant association was observed for 2006. The exact mechanism that contribute to these mixed findings in the present study is unknown. The best approach to address this discrepancy could be to conduct a prospective study in which each participant carry a portable air pollutant monitor daily over the study duration for direct exposure measurement and correlation with the onset of disease. Other confounding factors such as exposure to second-hand smoke, genetic differences, socioeconomic status, diet, lifestyle, age, physical activities etc. can be captured and recorded accurately for adjustment during the data analysis. The adjusted ORs of NO_2_ and NO_x_ for 2011 in the present study were 1.12 and 1.11 respectively. Similar ORs of the pollutants and prevalence of diabetes (1.011 and 1.015) were reported by Renzi et al. [44]. NO_2_ is one of the major components of traffic-related air pollution. Balti et al. [46] reported significant association of NO_2_ and diabetes (13% higher risk). The risk of insulin resistance for children who are exposed to traffic-related air pollution increased by 17.0 and 18.7% for every 2 standard deviations increase in ambient NO_2_ and particulate matter ≤10 μm in diameter respectively [47]. Similarly, Brook et al. [48] reported an association of NO_2_ exposure with higher diabetes risk in women (odds ratio of 1.04) but not in men at Hamilton and Toronto. Dijkema et al. [49] observed significant increased odds ratios of 1.48 and 1.44 for NO_2_ and when living within 250 m of a main road respectively for diabetes in Netherlands. Eze et al. [50] reviewed that both PM_2.5_ (OR = 1.10) and NO_2_ (OR = 1.08) increased the risk of developing diabetes mellitus by 8–10% per 10 μg/m^3^ exposure respectively.

The sample size for NHMS 2006 was larger compared with NHMS 2011 and NHMS 2015. Logistic regression model is used to calculate the odds ratio and adjust for confounders in the present study. Odds ratio determined for NHMS 2006 will converge to a better representation of normally distributed measures of association between an air pollution exposure and an outcome (diabetes prevalence) as the sample size increases. Larger sample size is generally required for studies with very common or extremely rare outcome, studies with higher number of exposure variables and characteristics, and studies with discrete exposures compared with continuous exposures [51]. As when the sample size increases, the confidence interval narrows substantially with better or more precise estimation of effects and less random errors.

Subsequent to modernisation and urbanisation, lifestyle changes from active to inactive sedentary lifestyle is likely to contribute to the growing burden of non-communicable diseases in developed and developing countries worldwide. There is a need to have a sensitive, reliable and validated assessment or surveillance tool for the capturing of different types, durations, intensities, levels and diversities of physical activities under different settings and living conditions at different countries. It is challenging to accurately measure habitual physical activity using currently available assessment tools that are developed and validated largely using Western populations with different cultural believes and practices compared with Asian populations. Currently, two questionnaires have been developed and validated as an international effort to provide comparable and standardise self-report measure of physical activity across countries i.e. Global Physical Activity Questionnaire (GPAQ) and International Physical Activity Questionnaire (IPAQ). GPAQ was developed by WHO for physical activity surveillance via face-to-face interviews conducted by trained interviewers in three settings (or domains i.e. activity at work, travel to and from places, recreational activities) and sedentary behaviour within a typical week (WHO) [52]. On the other hand, IPAQ captures overall physical activity according to intensity (high, moderate, low) as metabolic equivalent task (MET) minutes per week and is not domain specific via self-reporting within the last 7 days. In the present study, GPAQ was used to collect the physical activity for the year 2006 while IPAQ was used for 2011 and 2015. Caution shall be exercised in the interpretation, owing to change of physical activity assessment tool from GPAQ to the IPAQ, as this may impact on the accuracy and precision of the collected data. Different measurement tools use slightly different approaches in collecting the data. Both of these methods are very subjective where they are based on perception-based awareness recall and may not reflect the real practice in life. These methods may have over-reporting issue and recall bias compared with those objective approaches that measure the intensity, amount, duration and frequency of actual physical activities practice in life, and follows by the recording of all measurement via observation by a trained observer using questionnaire checklist. GPAQ combines both work and household activities together during assessment and analysis while IPAQ has separate domains for both activities. GPAQ focuses on physical activities in a typical week while IPAQ focuses on activities of the last 7 days. GPAQ classifies walking under moderate intensity activity while IPAQ assesses walking separately based on MET minutes. Au et al. [53] reported GPAQ may underestimated the total physical activities by not seeking domestic household activities separately from work-related activities. GPAQ was reported to have better reliability for people with stable work patterns than those with more variable physical activity.

Several limitations of this study are recognised and hence its findings should be interpreted with caution. First, this study could not completely exclude exposure measurement errors because the spatial resolution of the five ambient air pollution data was not very high, at the state-level, due to the lack of individual information on the time-location activity patterns. Hence, participants residing in different regions of the same state were assigned to the same level of air pollution exposure. Second, key potential confounding factors, such as dietary intake, have not been adjusted in the present models due to unavailability of such information. Therefore, it is not possible to infer causation but association between the long-term air pollution exposure and diabetes. Third, this study only investigated the association between diabetes prevalence and air pollutants with a rather limited population size and risk factors compared with other more pertinent endpoints in other longitudinal studies (diabetes incidence) or biomarkers (cross-sectional studies). Forth, in the present study, annual exposure estimates to air pollutants over the survey periods of 9 years was hypothesised to be representative for lifelong exposure based on the assumptions that no movement of the individual to another location within the study duration, no change of occupation which may confound the exposure to these air pollutants, no change of infrastructures such as additional housing areas, road transport and deforestation etc. This is one of the limitation of the present study and can be further improved if the study could correlate the onset of diabetes of each participant to the air pollution exposure on that particular year of disease onset with the adjustment of all the changes mentioned. Changes to any of those factors mentioned earlier may lead to exposure misclassification and can be further addressed using sensitivity test.

## Conclusion

The annual air pollution data showed an increase in the concentration of O_3_ throughout the investigated years from 2006 to 2015 for underdiagnosed diabetes. This unique analysis of the nation-wide series of NHMS survey data suggested that the exposure to O_3_, NO_2_, NO_x_ and PM_10_ may have mixed effect estimates towards the risk of DM and their roles are yet to be investigated further with direct measurement of pollutant exposure and adjusted with most if not all confounding factors with other interaction models. Despite of this, in the interest of public health in relation to the escalating increase in non-communicable diseases in Malaysia, there is an urgent need to reduce the concentrations of air pollutants, particularly O_3_, PM_10_, NO_2_ and NO_x._

## Supplementary information

**Additional file 1.**

## Data Availability

The datasets used in this study are available from the corresponding author upon reasonable request.

## Abbreviations

BMI: Body mass index
CAQM: Continuous Air Quality Monitoring
CI: Confidence intervals
DM: Diabetes mellitus
DOE: Department of Environment
EBs: Enumeration Blocks
GPAQ: Global Physical Activity Questionnaire
IDF: International Diabetes Federation
IPAQ: International Physical Activity Questionnaire
IQR: Interquartile range
LQs: Living Quarters
MET: Metabolic Equivalent of Task
NHMS: National Health and Morbidity Survey
NO_2_: Nitrogen dioxide
NO_x_: Nitrogen oxide
O_3_: Ozone
OR: Odds ratios
PM: Particular matters
SO_2_: Sulphur dioxide
T2DM: Diabetes mellitus type 2
WHO: World Health Organisation

## References

[1] International Diabetes Federation (IDF). 2017. https://diabetesatlas.org/resources/2017-atlas.html.

[2] Institute for Public Health, Ministry of Health (2000). National Health Morbidity Survey 1986–1987. Diabetes Mellitus.

[3] Institute for Public Health, Ministry of Health (2015). National Health Morbidity Survey, 2015a. Non-communicable diseases, risk factors & other health problems.

[4] Lim KG (2016). A review of adult obesity research in Malaysia. Med J Malaysia.

[5] Nolan CJ, Damm P, Prentki M (2011). Type 2 diabetes across generations: from pathophysiology to prevention and management. Lancet..

[6] Agardh E, Allebeck P, Hallqvist J, Moradi T, Sidorchuk A (2011). Type 2 diabetes incidence and socio-economic position: a systematic review and meta-analysis. Int J Epidemiol.

[7] Dendup T, Feng X, Clingan S, Astell-Burt T (2018). Environmental risk factors for developing type 2 diabetes mellitus: a systematic review. Int J Environ Res Public Health.

[8] Mezuk B, Li X, Cederin K, Rice K, Sundquist J, Sundquist K (2016). Beyond access: characteristics of the food environment and risk of diabetes. Am J Epidemiol.

[9] Alderete TL, Habre R, Toledo-Corral CM, Berhane K, Chen Z, Lurmann FW, Weigensberg MJ, Goran MI, Gilliland FD (2017). Longitudinal associations between ambient air pollution with insulin sensitivity, β-cell function, and adiposity in Los Angeles Latino children. Diabetes..

[10] Alderete TL, Chen Z, Toledo-Corral CM, Contreras ZA, Kim JS, Habre R, Chatzi L, Bastain T, Breton CV, Gilliland FD (2018). Ambient and traffic-related air pollution exposures as novel risk factors for metabolic dysfunction and type 2 diabetes. Curr Epidemiol Rep.

[11] Eze IC, Schaffner E, Fischer E, Schikowski T, Adam M, Imboden M, Tsai M, Carballo D, von Eckardstein A, Kunzli N, Schindler C, Probst-Hensch N (2014). Long-term air pollution exposure and diabetes in a population-based Swiss cohort. Environ Int.

[12] Eze IC, Imboden M, Kumar A, von Eckardstein A, Stolz D, Gerbase MW, Kunzli N, Pons M, Kronenberg F, Schindler C, Probst-Hensch N (2016). Air pollution and diabetes association: modification by type 2 diabetes genetic risk score. Environ Int.

[13] Kramer U, Herder C, Sugiri D, Strassburger K, Schikowski T, Ranft U, Rathmann W (2010). Traffic-related air pollution and incident type 2 diabetes: results from the SALIA cohort study. Environ Health Perspect.

[14] Peters A (2012). Air pollution and mortality from diabetes mellitus. Nat Rev Endocrinol.

[15] Afroz R, Hassan MN, Ibrahim NA (2003). Review of air pollution and health impacts in Malaysia. Environ Res.

[16] Mahiyuddin WR, Sahani M, Aripin R, Latif MT, Thach TQ, Wong CM (2013). Short-term effects of daily air pollution on mortality. Atmos Environ.

[17] Brauer M, Jamal HH (1998). Fires in Indonesia: crisis and reaction. Environ Sci Technol.

[18] Othman J, Sahani M, Mahmud M, Ahmad MK (2014). Transboundary smoke haze pollution in Malaysia: inpatient health impacts and economic valuation. Environ Pollut.

[19] Awang MB, Jaafar AB, Abdullah AM, Ismail MB, Hassan MN, Abdullah R, Johan S, Noor H (2000). Air quality in Malaysia: Impacts, management issues and future challenges. Respirology.

[20] Faiz A, Weaver CS, Walsh MP. Air pollution from motor vehicles: Standards and technologies for controlling emissions: The World Bank Washington, D.C.; 1996. 10.1596/0-8213-3444-1.

[21] Institute for Public Health, Ministry of Health. National Health Morbidity Survey, 2006. Institute for Public Health, Ministry of Health, Kuala Lumpur, Malaysia. http://www.iku.gov.my/index.php/research-eng/list-of-research-eng/iku-eng/nhms-eng/nhm s-2006-eng.

[22] Institute for Public Health, Ministry of Health. National Health Morbidity Survey, 2011. Methodology and General Findings. Vol. 1 Malaysia: Institute for Public Health, Ministry of Health, Kuala Lumpur, Malaysia. http://www.iku.gov.my/images/IKU/Document/ REPORT/NHMS2011-VolumeI.pdf.

[23] Institute for Public Health, Ministry of Health. National Health Morbidity Survey, 2015. Methodology and General Findings. Vol. 1 Malaysia: Institute for Public Health, Ministry of Health, Kuala Lumpur, Malaysia. http://www.iku.gov.my/images/IKU /Document/REPORT/ nhmsreport2015vol1.pdf.

[24] Fuziah MZ, Hong JYH, Wu LL, Fatimah H, Rahmah R, Muhammad Yazid J, Jamaiyah H, Jaya Purany SP, Mohamad Adam B, Permaa S (2012). 2nd Report of Diabetes in Children and Adolescents Registry (DiCARE) 2006–2008.

[25] World Health Organisation, WHO/NCD/NCS/99.2 (1999). Definition, diagnosis and classification of diabetes mellitus and its complications. Part 1: diagnosis and classification of diabetes mellitus. Report of a WHO Consultation.

[26] World Health Organisation (2000). Obesity: Preventing and managing the global epidemic. Report of a WHO Consultation on Obesity.

[27] Department of Environment, Ministry of Natural Resources and Environment (2013). Malaysia Environmental Quality Report (EQR).

[28] O’Donovan G, Chudasama Y, Grocock S, Leigh R, Dalton AM, Gray LJ, Yates T, Edwardson C, Hill S, Henson J, Webb D, Khunti K, Davies MJ, Jones AP, Bodicoat DH, Wells A (2017). The association between air pollution and type 2 diabetes in a large cross-sectional study in Leicester: the CHAMPIONS study. Environ Int.

[29] Strak M, Janssen N, Beelen R, Schmitz O, Vaartjes I, Karssenberg D, van den Brink C, Bots ML, Dijst M, Brunekreef B, Hoek G (2017). Long-term exposure to particulate matter, NO_2_ and the oxidative potential of particulates and diabetes prevalence in a large national health survey. Environ Int.

[30] Brook RD, Cakmak S, Turner MC, Brook JR, Crouse DL, Peters PA, van Donkelaar A, Villeneuve PJ, Brion O, Jerrett M, Martin RV, Rajagopalan S, Goldberg MS, Pope CA, Burnett RT (2013). Long-term fine particulate matter exposure and mortality from diabetes in Canada. Diabetes Care.

[31] Chen H, Burnett RT, Kwong JC, Villeneuve PJ, Goldberg MS, Brook RD, van Donkelaar A, Jerrett M, Martin RV, Brook JR, Copes R (2013). Risk of incident diabetes in relation to long-term exposure to fine particulate matter in Ontario, Canada. Environ Health Perspect.

[32] Puett RC, Hart JE, Schwartz J, Hu FB, Liese AD, Laden F (2011). Are particulate matter exposures associated with risk of type 2 diabetes?. Environ Health Perspect.

[33] Park SK, Wang W (2014). Ambient air pollution and type 2 diabetes: A systematic review of epidemiologic research. Curr Environ Health Rep.

[34] Liang F, Yang X, Liu F, Li J, Xiao Q, Chen J, Liu X, Cao J, Shen C, Yu L, Lu F, Wu X, Zhao L, Wu X, Li Y, Hu D, Huang J, Liu Y, Lu X, Gu D (2019). Long-term exposure to ambient fine particulate matter and incidence of diabetes in China: a cohort study. Environ Int.

[35] Kim KH, Kabir E, Kabir S (2015). A review on the human health impact of airborne particulate matter. Environ Int.

[36] Pearson JF, Bachireddy C, Shyamprasad S, Goldfine AB, Brownstein JS (2010). Association between fine particulate matter and diabetes prevalence in the U.S. Diabetes Care.

[37] Liu C, Yang C, Zhao Y, Ma Z, Bi J, Liu Y, Meng X, Wang Y, Cai J, Chen R, Kan H (2016). Associations between long-term exposure to ambient particulate air pollution and type 2 diabetes prevalence, blood glucose and glycosylated hemoglobin levels in China. Environ Int.

[38] Cantonea L, Iodicea S, Tarantinia L, Albettia B, Restellib I, Vignab L, Bonzini M, Pesatori AC, Bollati V (2017). Particulate matter exposure is associated with inflammatory gene methylation in obese subjects. Environ Res.

[39] Peng C, Bind MAC, Colicino E, Kloog I, Byun HM, Cantone L, Trevisi L, Zhong J, Brennan K, Dereix AE, Vokonas PS, Coull BA, Schwartz JD, Baccarelli AA (2016). Particulate air pollution and fasting blood glucose in non-diabetic individuals: associations and epigenetic mediation in the normative aging study, 2000-2011. Environ Health Perspect.

[40] Li H, Bai H, Yang C, Chen R, Wang C, Zhao Z, Kan H (2017). Acute effects of ambient temperature and particulate air pollution on fractional exhaled nitric oxide: a panel study among diabetic patients in Shanghai, China. J Epidemiol.

[41] Krishnan RM, Adar SD, Szpiro AA, Jorgensen NW, van Hee VC, Barr RG, O’Neill MS, Herrington DM, Polak JF, Kaufman JD (2012). Vascular responses to long- and short-term exposure to fine particulate matter MESA air (multi-ethnic study of atherosclerosis and air pollution). J Am Coll Cardiol.

[42] Jerrett M, Brook R, White LF, Burnett RT, Yu J, Su J, Seto E, Marshall J, Palmer JR, Rosenberg L, Coogan PF (2017). Ambient ozone and incident diabetes: A prospective analysis in a large cohort of African American women. Environ Int.

[43] Vella RE, Pillon NJ, Zarrouki B, Croze ML, Koppe L, Guichardant M, Pesenti S, Chauvin MA, Rieusset J, Géloën A, Soulage CO (2015). Ozone exposure triggers insulin resistance through muscle c-Jun N-terminal kinase activation. Diabetes.

[44] Renzi M, Cerza F, Gariazzo C, Agabiti N, Cascini S, Di Domenicantonio R, Davoli M, Forastiere F, Cesaroni G (2018). Air pollution and occurrence of type 2 diabetes in a large cohort study. Environ Int.

[45] Bass V, Gordon CJ, Jarema KA, MacPhail RC, Cascio WE, Phillips PM, Ledbetter AD, Schladweiler MC, Andrews D, Miller D, Doerfler DL, Kodavanti UP (2013). Ozone induces glucose intolerance and systemic metabolic effects in young and aged brown Norway rats. Toxicol Appl Pharmacol.

[46] Balti EV, Echouffo-Tcheugui JB, Yako YY, Kengne AP (2014). Air pollution and risk of type 2 diabetes mellitus: a systematic review and meta-analysis. Diabetes Res Clin Pract.

[47] Thiering E, Cyrys J, Kratzsch J, Meisinger C, Hoffmann B, Berdel D, von Berg A, Koletzko S, Bauer CP, Heinrich J (2013). Long-term exposure to traffic-related air pollution and insulin resistance in children: results from the GINIplus and LISAplus birth cohorts. Diabetologia..

[48] Brook RD, Jerrett M, Brook JR, Bard RL, Finkelstein MM (2008). The relationship between diabetes mellitus and traffic-related air pollution. J Occup Environ Med.

[49] Dijkema MB, Mallant SF, Gehring U, van den Hurk K, Alssema M, van Strien RT, Fischer PH, Nijpels G, Stehouwer CD, Hoek G, Dekker JM, Brunekreef B (2011). Long-term exposure to traffic-related air pollution and type 2 diabetes prevalence in a cross-sectional screening-study in the Netherlands. Environ Health.

[50] Eze IC, Hemkens LG, Bucher HC, Hoffmann B, Schindler C, Künzli N, Schikowski T, Probst-Hensch NM (2015). Association between ambient air pollution and diabetes mellitus in Europe and North America: systematic review and meta-analysis. Environ Health Perspect.

[51] Nemes S, Jonasson JM, Genell A, Steineck G (2009). Bias in odds ratios by logistic regression modelling and sample size. BMC Med Res Methodol.

[52] World Health Organisation. Global Physical Activity Questionnaire (GPAQ) analysis guide. Surveillance and population-based prevention of non-communicable diseases Department World Health Organisation, Switzerland. https://www.who.int/ncds/surveillance/steps/ resources/GPAQ_Analysis_Guide.pdf. Accessed 19 Sept 2019.

[53] Au TB, Blizzard L, Schmidt M, Pham LH, Magnussen C, Dwyer T (2010). Reliability and validity of the global physical activity questionnaire in Vietnam. J Physical Activity Health.

