# Supplementary material for: Association between long-term exposure to ambient air pollution and prevalence of diabetes mellitus among Malaysian adults
Source: Environ Health. 2020 Apr 3;19:37. doi: 10.1186/s12940-020-00579-w (PMC7119016; doi:10.1186/s12940-020-00579-w)
Supplement: Supplementary file 1 — Additional file 1. [file 12940_2020_579_MOESM1_ESM.docx]

**Supplemental materials**

1. NO_x_

1. NO_2_

75 µg/m^3^

1. SO_2_

90 µg/m^3^

1. O_3_
2. PM_10_

120 µg/m^3^

Supplementary Fig. 1. Annual average levels of (a) NO_x_, (b) NO_2_, (c) SO_2_, (d) O_3_ and (e) PM_10_ in ambient air of Malaysia from 2006 to 2015 in different states and Federal Territories (Kuala Lumpur/Putrajaya/Labuan). Blue lines indicate the recommended Malaysia Air Quality Guidelines (2018) values of the pollutants at µg/m^3^ per day (24 hours) except for O_3_ and NO_x_. There were no recommended Malaysia Air Quality Guidelines (2018) values of µg/m^3^ per day (24 hours) for O_3_ and NOx. For O_3_, the recommended value was only available for 8 hours of 120 µg/m^3^.

(Note: This figure should be printed in colour.)


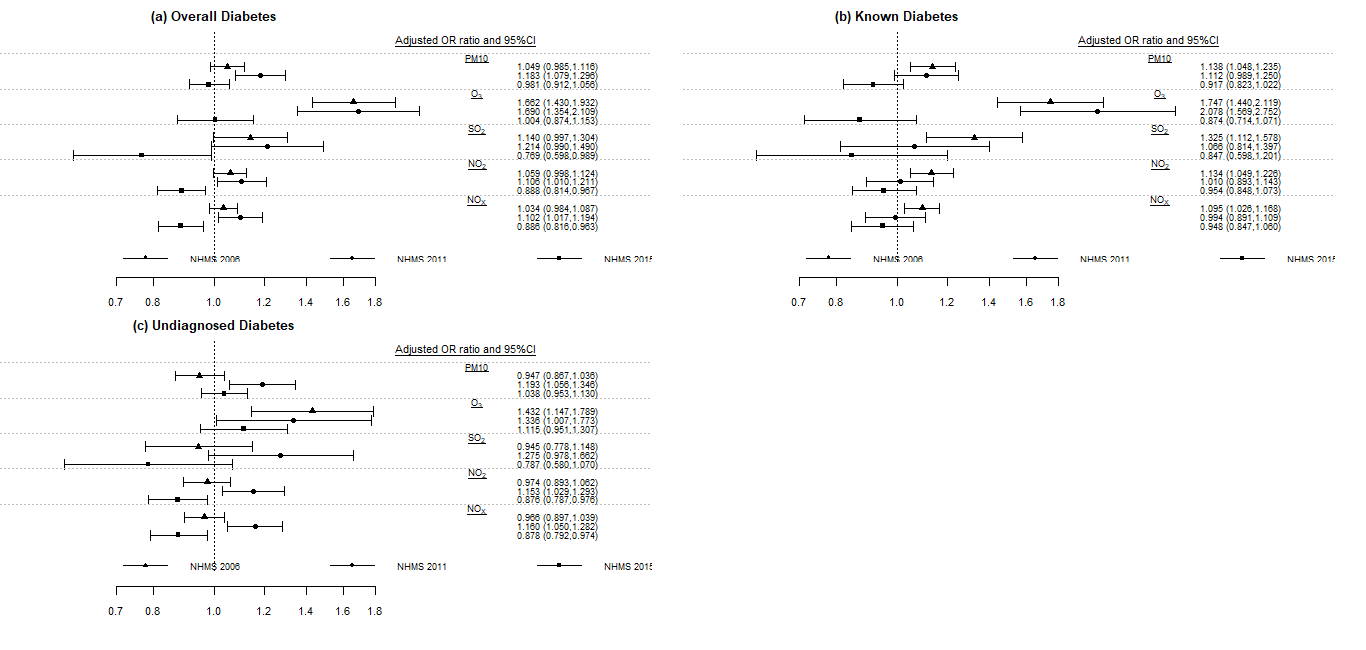


Supplementary Fig. 2. Odds ratio and 95% CIs between diabetes and annual average levels for air pollution exposures by NHMS survey years after excluding those who are living outside 50 km radius from the respective air monitoring stations (sensitivity analysis). Note: The ambient air pollution was modelled with the interquartile range increment (IQR) for the annual average levels of air pollutant exposures: IQR of NO_x_ = 16.83 μg/m^3^; NO_2_ = 9.57 μg/m^3^; SO_2_ = 4.16 μg/m^3^; O_3_ = 7.83 μg/m^3^; PM_10_ = 10.34 μg/m^3^. The models were adjusted for age, gender, ethnicity, urban/rural areas, body mass index (BMI) and physical activity.

**Supplementary Table 1**

| State | Area (km^2^) | Population size (million) | No of Station | Site_Id | Site_Location | Latitude | Longitude | Type | Percentage of residents with home distance between the station of | | |
| --- | --- | --- | --- | --- | --- | --- | --- | --- | --- | --- | --- |
|  |  |  |  |  |  |  |  |  | <= 10 km | <= 50 km | <= 100 km |
| Selangor | 7,931 | 6.18 | 5 | CA0011 | Sek. Men. (P) Raja Zarina, Kelang | 3.010333 | 101.408067 | Urban | 12.3 | 95.0 | 100.0 |
|  |  |  |  | CA0016 | Sek. Keb. Bandar Utama, Petaling Jaya | 3.110200 | 101.704567 | Industrial |  |  |  |
|  |  |  |  | CA0025 | Sek. Keb. TTDI Jaya, Shah Alam | 3.104783 | 101.556133 | Urban |  |  |  |
|  |  |  |  | CA0048 | Sekolah Menengah Sains, Kuala Selangor | 3.326533 | 101.258867 | Sub-urban |  |  |  |
|  |  |  |  | CA0060 | Kolej MARA, Banting | 2.816683 | 101.623017 | Sub-urban |  |  |  |
| Terengganu | 12,959 | 1.16 | 3 | CA0002 | Sek. Ren. Keb. Bukit Kuang, Teluk Kalung, Kemaman | 4.271000 | 103.430433 | Industrial | 18.3 | 56.3 | 90.3 |
|  |  |  |  | CA0024 | Kuarters TNB, Paka-Kertih | 4.598000 | 103.434933 | Industrial |  |  |  |
|  |  |  |  | CA0034 | Sek. Keb. Chabang Tiga, Kuala Terengganu | 5.307583 | 103.120217 | Urban |  |  |  |
| Perak | 21,038 | 2.47 | 5 | CA0008 | Sek. Men. Jalan Tasek, Ipoh | 4.629683 | 101.116067 | Industrial | 17.7 | 54.7 | 96.0 |
|  |  |  |  | CA0020 | Sek. Men. Keb. Air Puteh, Taiping | 4.899000 | 100.679700 | Industrial |  |  |  |
|  |  |  |  | CA0041 | Pejabat Pentadbiran Daerah Manjung, Perak | 4.200633 | 100.664017 | Sub-urban |  |  |  |
|  |  |  |  | CA0045 | Universiti Pendidikan Sultan Idris, Tanjung Malim | 3.687783 | 101.524433 | Sub-urban |  |  |  |
|  |  |  |  | CA0046 | Sek. Men. Pegoh, Ipoh, Perak | 4.552583 | 101.080933 | Urban |  |  |  |
| Pahang | 35,840 | 1.61 | 3 | CA0007 | Pej. Kajicuaca Batu Embun, Jerantut | 3.970633 | 102.347717 | Background | 13.0 | 48.3 | 68.0 |
|  |  |  |  | CA0014 | Sek. Keb. Indera Mahkota, Kuantan | 3.818967 | 103.296950 | Sub-urban |  |  |  |
|  |  |  |  | CA0015 | Sek. Keb. Balok Baru, Kuantan | 3.962100 | 103.382583 | Industrial |  |  |  |
| Pulau Pinang | 1,032 | 1.7 | 3 | CA0003 | Sek. Keb. Cederawasih, Taman Inderawasih, Perai | 5.391167 | 100.386883 | Industrial | 11.3 | 82.3 | 96.7 |
|  |  |  |  | CA0009 | Sek. Keb. Seberang Jaya II, Perai | 5.398167 | 100.403233 | Sub-urban |  |  |  |
|  |  |  |  | CA0038 | Universiti Sains Malaysia, Pulau Pinang | 5.358800 | 100.297733 | Sub-urban |  |  |  |
| N. Sembilan | 6,686 | 1.09 | 3 | CA0010 | Tmn. Semarak (Phase II), Nilai | 2.820767 | 101.814617 | Industrial | 26.3 | 66.3 | 100.0 |
|  |  |  |  | CA0047 | Sek. Men. Teknik Tuanku Jaafar, Ampangan, Seremban | 2.723633 | 101.968417 | Urban |  |  |  |
|  |  |  |  | CA0056 | Pusat Sumber Pendidikan N.S. Port Dickson | 2.440967 | 101.865933 | Urban |  |  |  |
| Melaka | 1652 | 0.89 | 2 | CA0006 | Sek. Men. Keb. Bukit Rambai | 2.265400 | 102.175900 | Industrial | 21.7 | 100.0 | 100.0 |
|  |  |  |  | CA0043 | Sek. Men. Tinggi Melaka, Melaka | 2.213150 | 102.234250 | Urban |  |  |  |
| Kelantan | 15,101 | 1.76 | 2 | CA0022 | Sek. Men. Keb. Tanjung Chat, Kota Bahru | 6.158667 | 102.250983 | Urban | 17.0 | 97.0 | 99.3 |
|  |  |  |  | CA0059 | SMK. Tanah Merah | 5.811183 | 102.133333 | Industrial |  |  |  |
| Kedah | 9,447 | 2.1 | 3 | CA0017 | Sek. Keb. Bakar Arang, Sungai Petani | 5.631433 | 100.469817 | Sub-urban | 11.7 | 77.3 | 99.3 |
|  |  |  |  | CA0032 | Komplek Sukan Langkawi, Kedah | 6.331717 | 99.858617 | Sub-urban |  |  |  |
|  |  |  |  | CA0040 | Sek. Men. Agama Mergong, Alor Setar | 6.136967 | 100.348000 | Urban |  |  |  |
| Putrajaya/Labuan | 92 | 0.09 | 4 | CA0042 | Taman Perumahan Majlis Perbandaran Labuan | 5.333000 | 115.238583 | Sub-urban | Together with Sabah | | |
|  | 49 | 0.08 |  | CA0053 | Sek. Keb. Putrajaya 8(2), Jln P8/E2, Presint 8, Putrajaya | 2.931917 | 101.681817 | Urban | 100.0 | 100.0 | 100.0 |
|  | 243 | 1.78 |  | CA0054 | Sek.Men.Keb.Seri Permaisuri, Cheras | 3.106267 | 101.717867 | Urban | 46.0 | 98.0 | 99.7 |
|  |  |  |  | CA0058 | Sek. Keb. Batu Muda, Batu Muda, Kuala Lumpur | 3.212467 | 101.682150 | Urban |  |  |  |
| Johor | 19,102 | 3.61 | 4 | CA0001 | Sek. Men. Pasir Gudang 2, Pasir Gudang | 1.470417 | 103.893950 | Industrial | 14.7 | 52.3 | 81.7 |
|  |  |  |  | CA0019 | Institut Perguruan Malaysia, Temenggong Ibrahim, Larkin, Johor Bharu | 1.496917 | 103.726950 | Industrial |  |  |  |
|  |  |  |  | CA0044 | Sek. Men. Teknik Muar, Muar, Johor | 2.061917 | 102.593117 | Sub-urban |  |  |  |
|  |  |  |  | CA0057 | SMA, Bandar Penawar, Kota Tinggi | 1.558333 | 104.221833 | Urban |  |  |  |
| Perlis | 818 | 0.25 | 1 | CA0033 | Institut Latihan Perindustrian (ILP) Kangar | 6.423733 | 100.184100 | Sub-urban | 24.3 | 91.7 | 100.0 |
| Sarawak | **124,451** | **2.619** | 10 | CA0031 | Dewan Suarah, Limbang | 4.758817 | 115.013550 | Sub-urban | 8.7 | 40.0 | 66.3 |
|  |  |  |  | CA0035 | Pejabat Daerah Samarahan, Kota Samarahan | 1.455133 | 110.491633 | Rural |  |  |  |
|  |  |  |  | CA0036 | Kompleks Sukan, Sri Aman | 1.240417 | 111.460483 | Sub-urban |  |  |  |
|  |  |  |  | CA0055 | Stadium Tertutup, Kapit | 2.014583 | 112.927333 | Rural |  |  |  |
|  |  |  |  | CA0061 | ILP MIRI | 4.490200 | 114.039350 | Rural |  |  |  |
|  |  |  |  | CA0004 | Medical Store, Kuching | 1.562233 | 110.388817 | Industrial |  |  |  |
|  |  |  |  | CA0026 | Ibu Pejabat Polis Sibu, Sibu | 2.314267 | 111.831767 | Sub-urban |  |  |  |
|  |  |  |  | CA0027 | Balai Polis Pusat Bintulu | 3.176450 | 113.040550 | Sub-urban |  |  |  |
|  |  |  |  | CA0028 | Sek. Men. Dato Permaisuri Miri | 4.424267 | 114.012183 | Sub-urban |  |  |  |
|  |  |  |  | CA0029 | Balai Polis Pusat Sarikei | 2.133200 | 111.522517 | Sub-urban |  |  |  |
| Sabah | 73,904 | 3.72 | 4 | CA0030 | Sek. Men. Keb Putatan, Tg Aru, Kota Kinabalu | 5.893717 | 116.043267 | Urban | 7.7 | 49.7 | 64.0 |
|  |  |  |  | CA0039 | Pejabat JKR Tawau, Sabah | 4.250267 | 117.9361 | Urban |  |  |  |
|  |  |  |  | CA0049 | Sek. Men. Keb Gunsanad, Keningau | 5.338550 | 116.162817 | Sub-urban |  |  |  |
|  |  |  |  | CA0050 | Pejabat JKR Sandakan, Sandakan | 5.864417 | 118.091317 | Sub-urban |  |  |  |
